# Processing of Calcium Magnesium Silicates by the Sol–Gel Route

**DOI:** 10.3390/gels8090574

**Published:** 2022-09-09

**Authors:** Andrada-Elena Alecu, Claudiu-Constantin Costea, Vasile-Adrian Surdu, Georgeta Voicu, Sorin-Ion Jinga, Cristina Busuioc

**Affiliations:** Department of Science and Engineering of Oxide Materials and Nanomaterials, Faculty of Chemical Engineering and Biotechnologies, University Politehnica of Bucharest, RO-060042 Bucharest, Romania

**Keywords:** silicates, diopside, akermanite, merwinite, sol–gel, tissue engineering

## Abstract

In this work, calcium magnesium silicate ceramics were processed through the sol–gel method in order to study the crystalline and morphological properties of the resulting materials in correlation with the compositional and thermal parameters. Tetraethyl orthosilicate and calcium/magnesium nitrates were employed as sources of cations, in ratios specific to diopside, akermanite and merwinite; they were further subjected to gelation, calcination (600 °C) and thermal treatments at different temperatures (800, 1000 and 1300 °C). The properties of the intermediate and final materials were investigated by thermal analysis, scanning electron microscopy, energy dispersive X-ray spectroscopy, Fourier transform infrared spectroscopy, X-ray diffraction and Rietveld refinement. Such ceramics represent suitable candidates for tissue engineering applications that require porosity and bioactivity.

## 1. Introduction

One of the most recent challenges for researchers in the scientific field is obtaining new biomaterials for tissue engineering. In this sense, bioceramics based on calcium magnesium silicates are increasingly studied, following their use in medicine due to their properties, such as high biocompatibility, bioactivity and biodegradability [1,2,3], superior mechanical properties [4,5,6] and appropriate degradability rate [7,8,9], being often compared with calcium silicates (CaSiO_3_) and calcium phosphates (Ca_3_(PO_4_)_2_) [10]. The class of calcium silicates also includes the ceramic components of the ternary system CaO–SiO_2_–MgO [11,12,13], such as diopside (CaMgSi_2_O_6_), akermanite (Ca_2_MgSi_2_O_7_) and merwinite (Ca_3_MgSi_2_O_8_). Their multifunctional properties recommend them as candidates for the development of materials suitable for the treatment of bone tissue injuries, as well as its regeneration [12,13,14,15,16,17]; this is due to Ca and Mg ions [1,18] that promote the process of mineralization through apatite deposition [3,19] and enhance cell proliferation and differentiation [1,20,21]. Some researchers prepared larnite and rankinite through the sol–gel combustion method [22], but also monticellite and diopside from eggshell waste via the combustion route [23], with good results in terms of mechanical strength, bioactivity, antibacterial activity, as well as cell adhesion, proliferation and differentiation. Sodium calcium silicate is another bioactive ceramic that was synthesized by the combustion technique and whose bioactivity was found to be rapid when compared with that of calcium silicates and calcium magnesium silicates [24].

There are several methods of preparing these silicates, such as sol–gel route [25,26], solid-state reaction [27,28,29], co-precipitation approach [10,30], and the spray pyrolysis technique [31,32], but the most used and the simplest one is the well-known wet-chemistry protocol that involves the transition from a sol to a gel, subsequently completed by a thermal treatment [32,33]. Sol–gel is a method that starts by mixing some precursors, either salts or alkoxides [34], continues with the pH adjustment and ends up with the achievement of a homogeneous and stable solution, which will undergo hydrolysis and gelation processes [7]. Usually, after the maturation stage of the transparent gel, a heating program is applied, determining the formation of predominantly white powders [35]. The main advantages of the sol–gel technique are the morphological control and good adhesion during deposition of thin films [30,33,36], as well as the uniform mixing of the precursors, which leads to the obtaining of homogeneous products at low temperatures [37] and, in addition, the powders when processed in such a way can be further sintered at high temperatures [38].

Recently, it was reported that diopside powder synthesized through the sol–gel method, followed by calcination at 950 °C, consists of particles with sizes ranging between 22 and 38 nm [39]. In the case of merwinite prepared by the sol–gel technique, at calcination temperatures of 850 or 1400 °C, the particle dimensions were between 25 nm and 3.5 μm [19,40]. By applying the same approach for akermanite, but with calcination at 1300 °C, values belonging to the 5–40 μm range were obtained [41].

Various papers showed that the field is also developing in the direction of doping these silicates with different types of oxides containing Sr, Zn, Cu, Ti, Zr, etc. metal ions to give them superior or new properties [8,28,42,43]. It was demonstrated that diopside, akermanite and merwinite powders can be integrated in composite scaffolds together with other materials, thus forming biomaterials such as: diopside/PLA [16], diopside/PCL [44], diopside/graphene [45], diopside/silk [46], akermanite/PLA [47], akermanite/PGLA [14], merwinite/PLGA [17], merwinite/PCL [48]. Likewise, many studies confirmed the ability of these three silicates to trigger the formation of a surface bone-like apatite and the high rate of biodegradability during exposure to biomimetic environments for several days. In the case of diopside and akermanite, it was observed that apatite crystals increase with soaking time (from 9 to 28 days) [5,10,49]; the significant increase in the content of Ca, Mg and Si ions in the simulated body fluid solution after immersion for 28 days indicated that merwinite can be hydrolyzed fast, providing a connection with the living bone and a high biodegradability [50].

Considering the great potential of calcium magnesium silicates in the field of medical applications, especially for the development of bone tissue substitutes or bone defect fillers, in this study, three different systems were designed starting from the compositions of diopside, akermanite and merwinite and subsequently produced in the form of thermally treated ceramic powders. These were characterized from multiple perspectives with the purpose of understanding the effects of different calcium content, which increases in the series: diopside < akermanite < merwinite. Both morphology and mineralogical composition were correlated with the processing parameters.

## 2. Results and Discussion

According to the definition of the sol–gel method, it implies the primary preparation of solutions or suspensions that are further processed in order to ensure the hydrolysis and polycondensation/polymerization of the constituent entities, followed by the occurrence of bridging oxygens and finally the formation of a three-dimensional structure with increasing viscosity as the gel ages; the latter is able to embed a large quantity of solvent, which opens new perspectives towards its subsequent processing. Thus, the gel can be converted into particles, fibers, film or scaffold. Moreover, the solvent content can be removed in a slow or fast manner, allowing completely different morphologies to be reached. Overall, the sol–gel approach ensures the obtaining of oxide powders with complex composition, high purity, increased homogeneity and last, but not least, small particle size.

### 2.1. Gels Characterization

**Figure 1:**
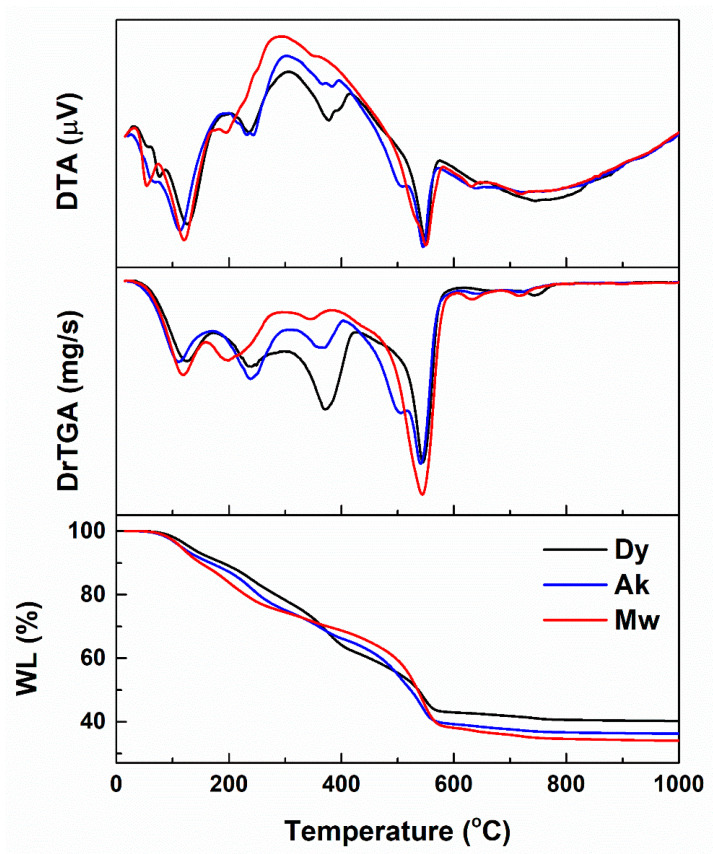
Complex thermal analyses of the dry gels corresponding to: diopside (Dy), akermanite (Ak) and merwinite (Mw) compositions. WL represents weight loss, DrTGA is the derivative thermogravimetric analysis and DTA stands for differential thermal analysis.

In the first part, three gels with compositions specific to Dy, Ak and Mw were obtained. These were transparent and bulky immediately after gelation, but white, contracted and cracked after drying. Small amounts of ground gels were subjected to complex thermal analysis, the resulting curves being available in Figure 1. Analyzing comparatively the thermogravimetric behavior (WL), it can be stated that the highest weight loss corresponds to Mw, namely 66%, followed by Ak with 64% and Dy with 60%. This can be explained based on the quantity of calcium nitrate added to the precursor solution, which increased in the series: Dy < Ak < Mw. As well, the mass loss is mainly recorded below the temperature of 600 °C, since the gas-generating components are taken out by volatilization, burning or decomposition at such temperatures [51]. After this value, the solid-state reactions take place and the reduction was insignificant (3–5%), which led to the selection of 600 °C as the calcination temperature. Otherwise, the general features revealed by this investigation technique are quite similar for all three specimens, with small shifts or variations in intensity that do not change the general appearance.

According to the DrTGA curves, there are four weight loss stages, highlighted by the function minima. Moreover, DTA curves indicate at least three endothermic processes and an exothermic one below 600 °C, if we do not take into account the shoulders or the peaks with low intensity. The first stage of mass loss occurs between 40 and 170 °C, which is associated with an endothermic process, probably attributed to the removal of residual solvents or adsorbed water. The second stage of mass loss takes place between 170 and 300 °C, which is linked with an endothermic process, mainly the elimination of chemically bound water (dehydration of recrystallized nitrates and their incipient decomposition). The third stage of mass loss occurs between 300 and 420 °C and is associated with an overlap of endothermic and exothermic processes, which could indicate the first phase of nitrate decomposition and the organic residue burning. The last stage of mass loss takes place between 420 and 600 °C and is linked with an endothermic process, certainly due to the completion of nitrate decomposition. At higher temperatures, between 600 and 800 °C, several weight variations occur, presumably generated by the accidental carbonation of some species in the sample with CO_2_ from the atmosphere, resulting in compounds that are weakly crystalline and decarbonate in this temperature range [52,53].

**Figure 2:**
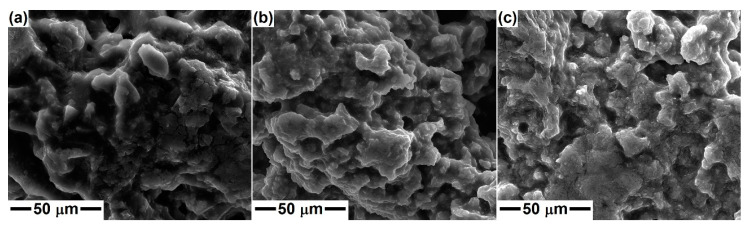
SEM images of the dry gels corresponding to: (**a**) diopside, (**b**) akermanite and (**c**) merwinite compositions.

Figure 2 shows the SEM images captured on the dry and ground gels with compositions specific to Dy, Ak and Mw. Irregularly branched formations with a specific morphology of polymeric material are visible; these consist of blocks with sizes between 5 and 50 μm. In places, granular entities embedded in a continuous matrix, uniform in size, can be detected. No important differences are discernible from one specimen to another because the morphology is induced at this stage by the 3D skeleton built on the oxygen bridges (Si–O–Si) arising after the hydrolysis and polycondensation/polymerization processes, with Ca and Mg ions trapped in this gel matrix.

### 2.2. Ceramics Characterization

**Figure 3:**
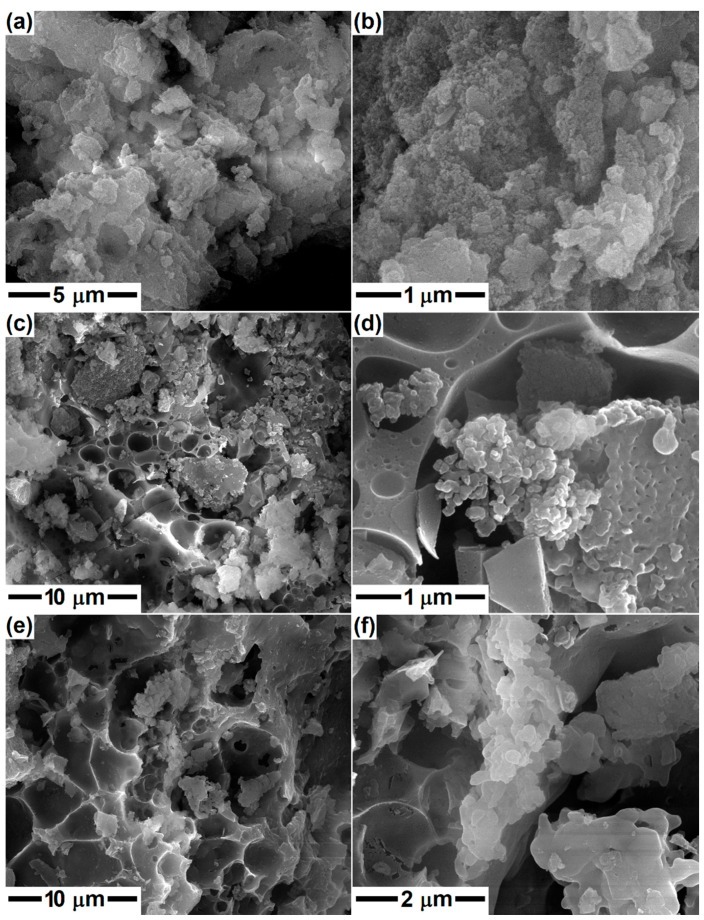
SEM images of the ceramics corresponding to diopside composition, thermally treated at: (**a**,**b**) 600 °C; (**c**,**d**) 800 °C; and (**e**,**f**) 1000 °C.

**Figure 4:**
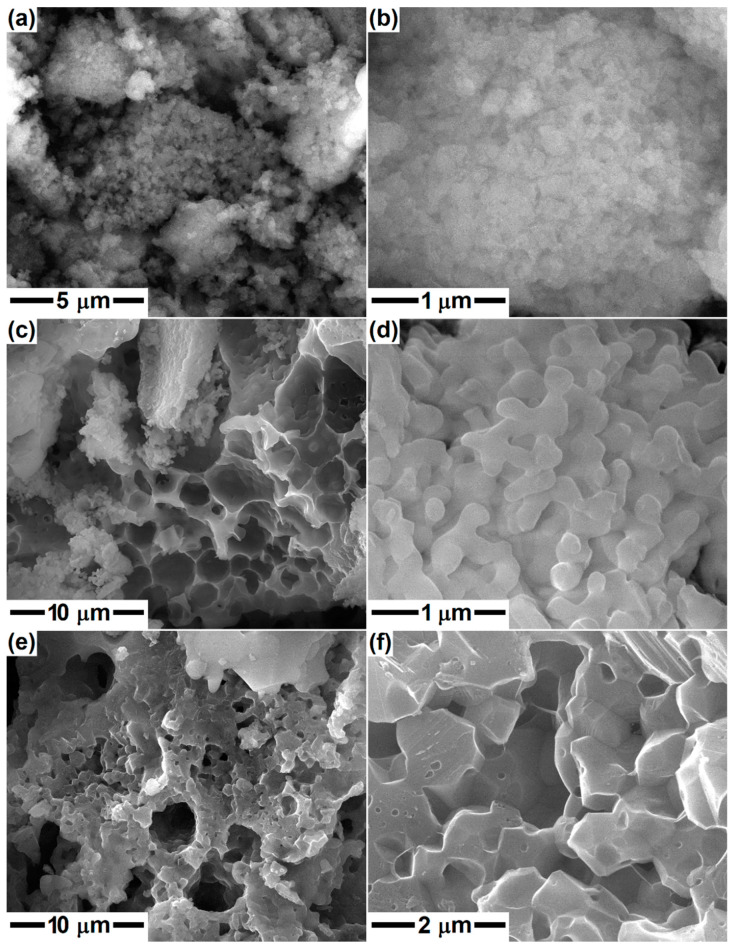
SEM images of the ceramics corresponding to akermanite composition, thermally treated at: (**a**,**b**) 600 °C; (**c**,**d**) 1000 °C; and (**e**,**f**) 1300 °C.

**Figure 5:**
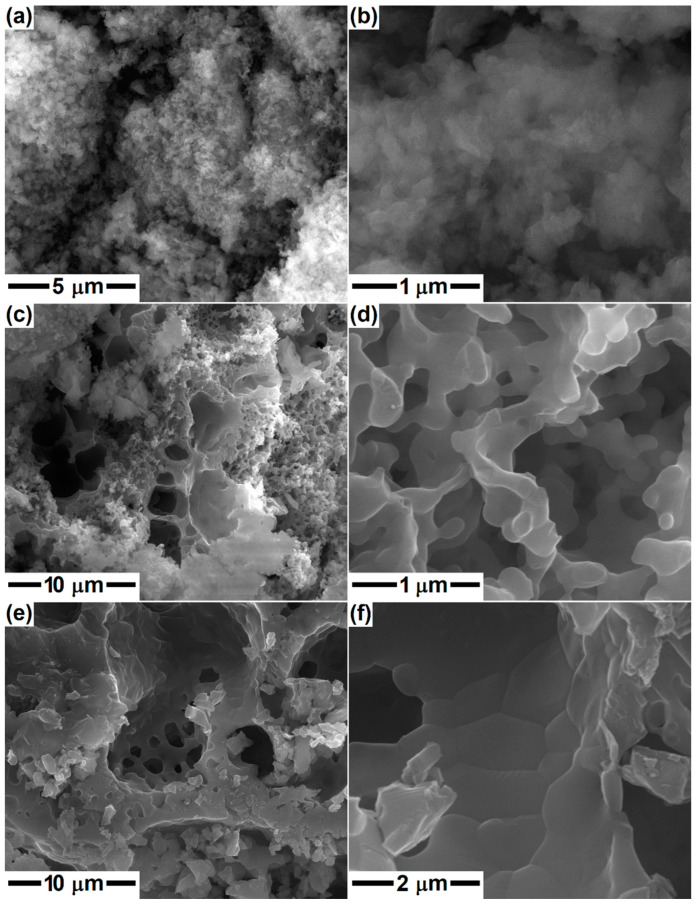
SEM images of the ceramics corresponding to merwinite composition, thermally treated at: (**a**,**b**) 600 °C; (**c**,**d**) 1000 °C; and (**e**,**f**) 1300 °C.

In the second part, the gels were calcined at 600 °C in order to remove the gas-generating constituents and possibly attain a preliminary crystallization of the silicate compounds. Afterward, the calcined powders were subjected to a second thermal treatment with the aim of studying the phase composition evolution at higher temperatures (800, 1000 and 1300 °C) and the implications of different calcium content. The morphology of the samples with compositions specific to Dy, Ak and Mw is displayed in Figure 3, Figure 4 and Figure 5. The SEM images at different magnifications evidence the presence of nanoparticles, which appear in the form of agglomerates with sizes between 1 and 5 μm in the case of calcination at 600 °C; in the second image in each case, individual particles with quasi-spherical shape, an average diameter of 25 nm and narrow size distribution can be identified. The temperature increase to 800 or 1000 °C leads to a different microstructure, in which the particles built sponge structures, having thin walls and encapsulating large pores. The maximum temperature (1300 °C) causes partial sintering of the powder, resulting in consolidated blocks with a high percentage of open porosity, especially in the case of Mw composition. Otherwise, the growth of particle/grain dimension with temperature increase is obvious, but also the distinct aspect of Ak-1300 and Mw-1300 samples: the first one presents well-packed faceted robust grains, while the second one consists of perfectly joint round grains of different sizes. Thus, the superior temperature promotes material diffusion and enables the initiation of sintering with positive repercussions on the mechanical properties of a material that works under permanent loads.

**Figure 6:**
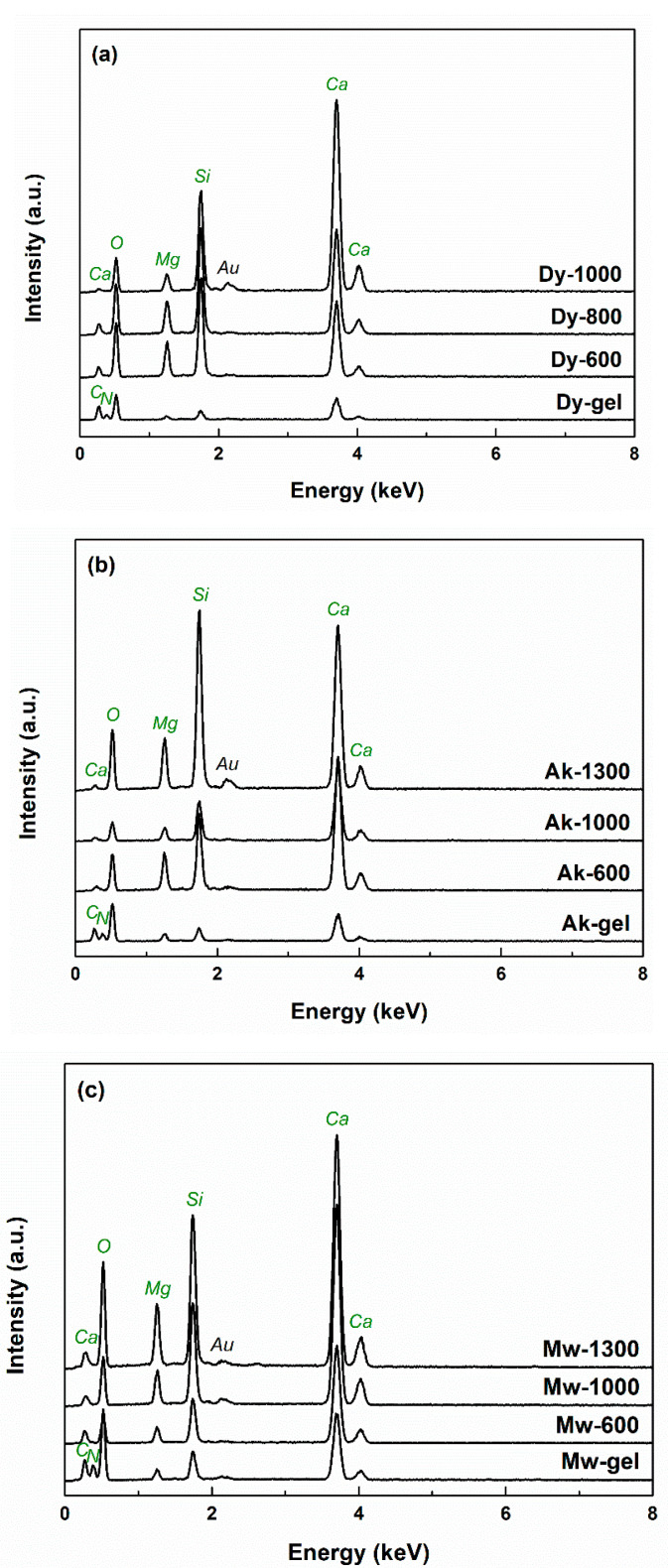
EDX spectra of the dry gels and ceramics corresponding to: (**a**) diopside; (**b**) akermanite; and (**c**) merwinite compositions.

The EDX spectra registered on the gels and powders provide information about the chemical elements present in each sample. Figure 6 centralizes all the data corresponding to the compositions of Dy, Ak and Mw. Maxima specific to all elements of interest (Ca, Mg, Si and O) can be observed, but also differences in intensity, probably related to local inhomogeneities. The presence of Au is justified by the deposition of a conductive layer on the sample’s surface before microscopy so that the quality of SEM images is high. The powders underwent a thermal treatment, which eliminated the gas-generating components, leaving clean EDX spectra; the presence of C and N is noticeable only for the gels due to the use of an alkoxide and two nitrate-type precursors.

**Figure 7:**
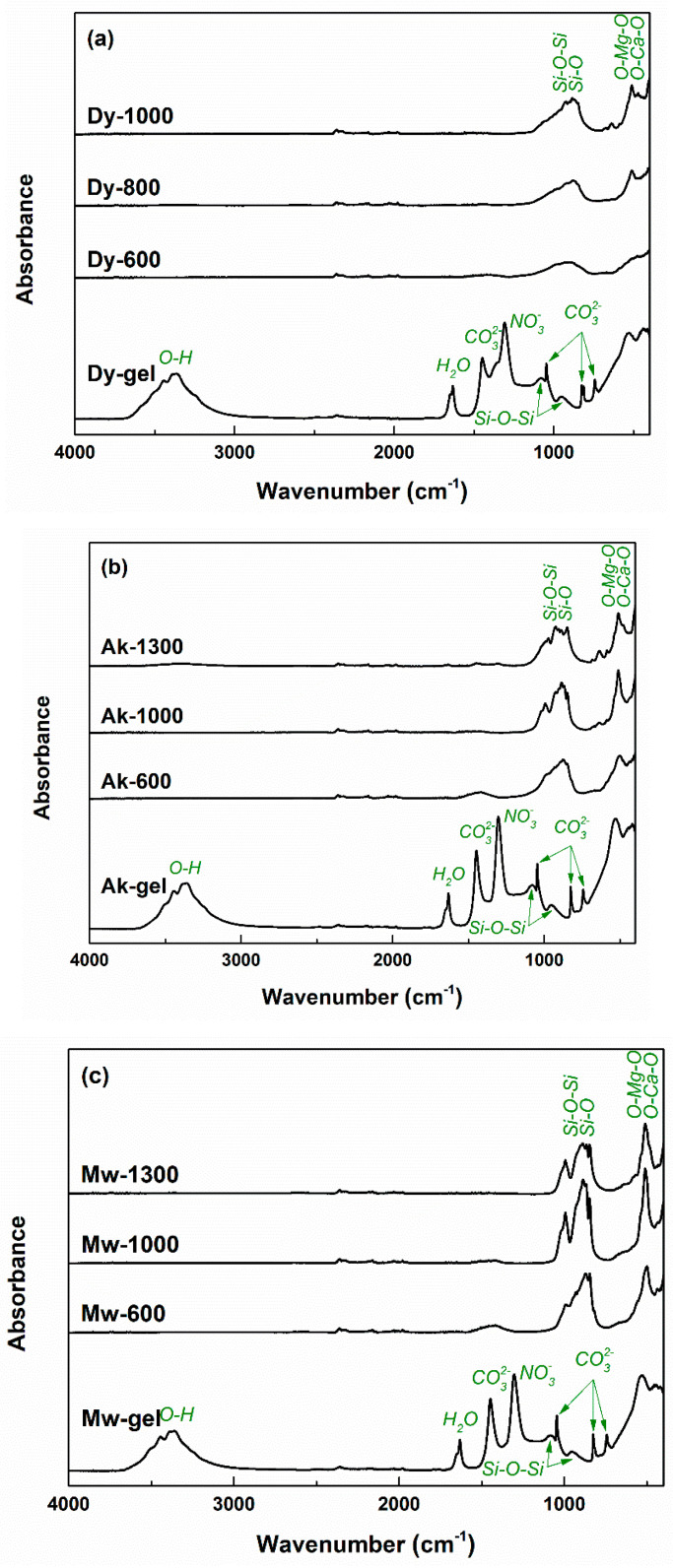
FTIR spectra of the dry gels and ceramics corresponding to: (**a**) diopside; (**b**) akermanite; and (**c**) merwinite compositions.

Figure 7 shows the FTIR spectra obtained on the gels and powders with compositions of Dy, Ak and Mw. Within the gels, the broad band located at around 3370 cm^−1^ is attributed to the stretching vibrations of adsorbed and bound water and OH^−^ groups, while the narrow one placed at around 1630 cm^−1^ is assigned to the bending vibrations of water. According to the scientific literature, around the values of 1450, 1050, 820 and 740 cm^−1^, the asymmetric and symmetric stretching, as well as the out-of-plane and in-plane bending vibrations of C–O bonds from CO_3_^2−^ groups, can be observed [33]; these were generated through the contamination with atmospheric CO_2_ during synthesis. The sharp band associated with the symmetric stretching vibrations of NO_3_^−^ groups originating from the nitrate-type precursors introduced into the precursor solution can be found around 1300 cm^−1^ [33].

It is well-known that the signals of Si–O bonds can be identified in the range of 800–1100 cm^−1^. If, in the FTIR spectra of the gel, there are two wide and low bands centred at around 1080 and 950 cm^−1^, associated with the stretching vibrations of Si–O–Si bonding, the situation is different in the FTIR spectra of the thermally treated ceramics: both Si–O–Si and Si–O bonding are visible, integrated into a complex band with multiple peaks; more precisely, antisymmetric and symmetric stretching vibrations of Si–O–Si bridging oxygen bonds in SiO_4_^4−^ tetrahedra, as well as Si–O–Ca and Si–O–Mg non-bridging oxygen bonds overlap and give rise to a broad and jagged band [53,54]. In the case of the signals that emerged below the value of 600 cm^−1^, the fingerprints of O–Ca–O and O–Mg–O bonding can be identified as bending vibrations at 510 and 540 cm^−1^, respectively; Mg–O contribution is sometimes integrated into Ca–O contribution or detected as a shoulder [55,56]. The bands seem to be better defined, with well-separated maxima when it goes from Dy to Ak and then to Mw, in strong correlation with the particularities of the crystalline structure and degree of ordering.

**Figure 8:**
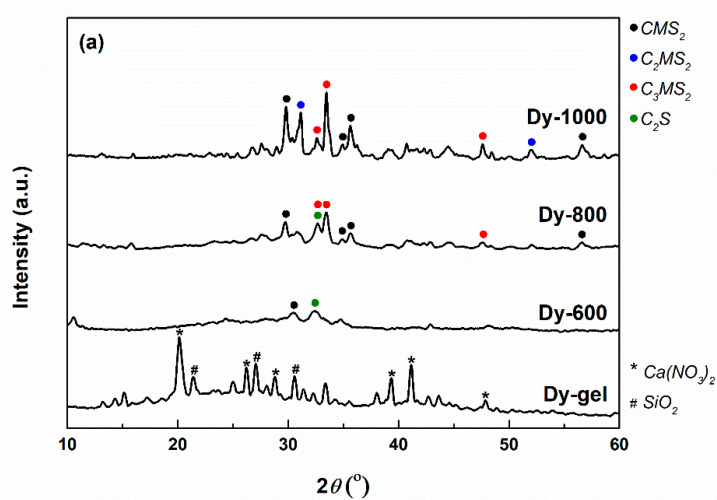

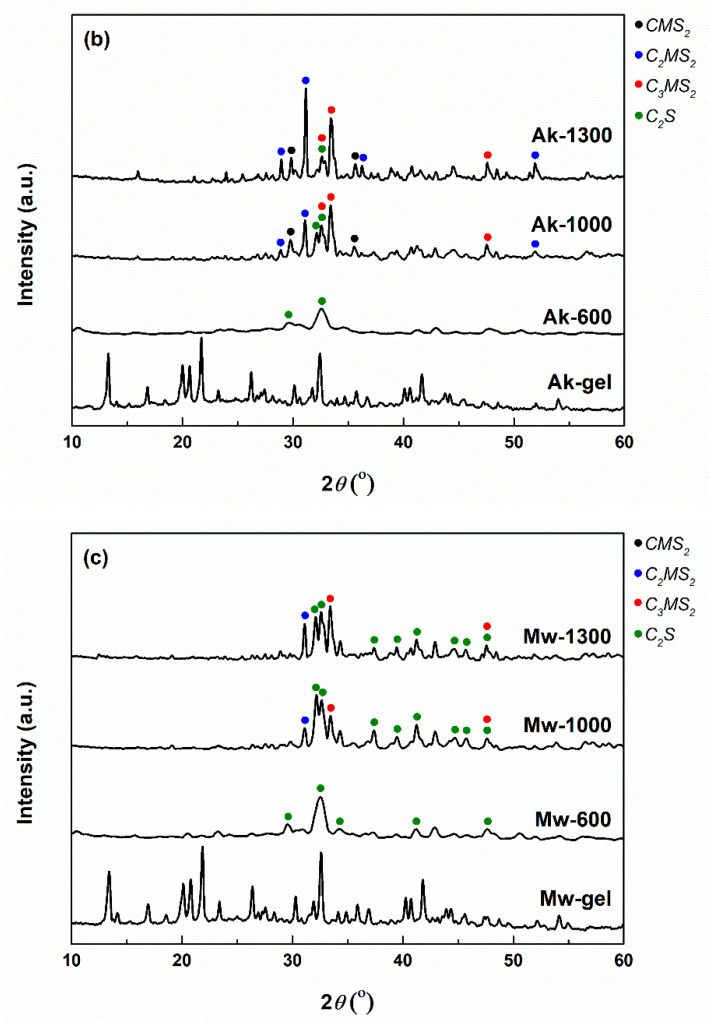
XRD patterns spectra of the dry gels and ceramics corresponding to: (**a**) diopside; (**b**) akermanite; and (**c**) merwinite compositions.

The mineralogical composition and type of crystalline structure were investigated with the help of XRD analysis. The corresponding XRD patterns are shown in Figure 8, for all three compositions (Dy, Ak and Mw). In the case of Dy, the evolution is obvious from the gel stage to the powder thermally treated at 1000 °C. Thus, the gel contains calcium nitrate (Ca(NO_3_)_2_, cubic crystal system, ICDD 00-007-0204) recrystallized from solution and different types of ordered silicon dioxide (SiO_2_, tetragonal crystal system—ICDD 00-081-1666, hexagonal crystal system—ICDD 00-083-2471, etc.), which turn into a less crystalline mass after calcination at 600 °C; the last mainly consists of a type of diopside (CaMgSi_2_O_6_, monoclinic crystal system, ICDD 00-083-1821) and a mixture of dicalcium silicates (Ca_2_SiO_4_, monoclinic crystal system—ICDD 00-083-0464 and orthorhombic crystal system—ICDD 00-083-2457). The heating at 800 °C promotes the crystallization of all three calcium magnesium silicates: another type of diopside (CaMgSi_2_O_6_, monoclinic crystal system, ICDD 00-083-1817), akermanite (Ca_2_MgSi_2_O_7_, tetragonal crystal system, ICDD 00-083-1815) and merwinite (Ca_3_MgSi_2_O_8_, monoclinic crystal system, ICDD 00-074-0382), but also the quantitative growth of dicalcium silicate previously mentioned. The temperature of 1000 °C seems to be the optimal temperature for the binary compound (Ca_2_SiO_4_) removal, since the associated ceramic presents only diopside, akermanite and merwinite, with increased crystallinity, as the high intensity and low width of the peaks indicate.

The XRD patterns of the samples with Ak and Mw compositions are quite similar in the stages of gel and 600 °C calcinated powders. In both cases, the crystalline phases from the gel could not be identified, probably because they are non-stoichiometric compounds. Then, the calcined samples exhibit a small crystallinity, conferred by dicalcium silicates formerly indicated. Going further, the two systems evolve slightly differently at higher temperatures (1000 and 1300 °C). In the case of Ak composition and the 1000 °C temperature, merwinite, followed by akermanite seem to be the major phases, accompanied by diopside and dicalcium silicate as minor phases; at 1300 °C, the same four crystalline compounds are maintained, but akermanite becomes predominant, a fact that was expected and desired since this composition was targeted. When it comes to Mw composition, the number of phases is reduced to three: akermanite, merwinite and dicalcium silicate; the temperature of 1000 °C favors the prevalent crystallization of dicalcium silicate, which changes at 1300 °C when merwinite and diopside increase quantitatively.

Concluding, the temperature increase ensures the rise of crystallinity degree, as well as the conversion of the primary or intermediate compounds to the desired ternary compounds (diopside, akermanite and merwinite) through a continuous change of phase ratio. The general reactions can be summarized as displayed below.

2 × C + S → C_2_S (1)

y × C_2_S + 2 × M + (4 − y) × S → 2 × C_y_MS_2_ (2)

A more detailed insight into the phase composition of the samples thermally treated at the highest temperatures (1000 and 1300 °C) was possible due to a Rietveld refinement on the recorded XRD patterns. The values obtained after performing this type of processing are listed in Table 1. Thus, the numbers confirm the previous statements regarding the ratios between different crystalline compounds. Indeed, the ceramic corresponding to Dy composition has diopside as the main ordered phase (almost 60%), followed by merwinite (about 30%) and akermanite (around 10%). All three ternary compounds (diopside, akermanite and merwinite) are present in fairly equivalent amounts in the sample with Ak composition. The behavior is completely different for the material corresponding to Mw composition, namely the quantitative superiority of dicalcium silicate (almost 60%), compared to merwinite (about 30%) and akermanite (around 10%).

It is obvious that all three oxide systems follow different pathways of crystallization even though the processing and thermal history are identical, calcium content being the determining parameter; this will also have important implications in the next stage when the biomineralization ability of these materials will be investigated.

## 3. Conclusions

Starting from diopside, akermanite and merwinite compositions and employing the sol–gel method, crystalline ceramics were obtained after applying thermal treatments at different temperatures. If the powders calcined at 600 °C were in an incipient state of crystallization, mainly given by the dicalcium silicate binary compound, the materials thermally treated at higher temperatures (800, 1000 and 1300 °C) displayed distinct phase compositions as a result of multiple and competitive solid-state reactions defined by specific thermodynamic conditions. Only the sample corresponding to Dy composition presented diopside as the leading crystalline compound, while Ak composition ended up as a balanced mixture of diopside, akermanite and merwinite and Mw composition exhibited a majority of dicalcium silicate.

The approached ceramics contain ternary compounds in the oxide system CaO–MgO–SiO_2_ and present tremendous potential as biomaterials in the field of hard tissue engineering. They are well-known as biocompatible and bioactive bioceramics, with some differences in terms of mineralization kinetics due to the different calcium content. Future research will be dedicated to the development of calcium magnesium silicate-based scaffolds with controlled properties, as well as to an extended biological evaluation of all the developed materials. Such silicate powders can be further processed by 3D printing, but only after a severe selection of the organic counterpart, necessary for ensuring appropriate rheological properties, as well as working parameters, essential for acquiring high-quality 3D porous materials.

## 4. Materials and Methods

### 4.1. Materials

Calcium nitrate tetrahydrate (Ca(NO_3_)_2_·4H_2_O, 99%, Sigma-Aldrich, Burlington, MA, USA), magnesium nitrate hexahydrate (Mg(NO_3_)_2_·6H_2_O, 99%, Sigma-Aldrich) and tetraethyl orthosilicate (Si(OC_2_H_5_)_4_, TEOS, 98%, Sigma-Aldrich) were used as cationic precursors, while nitric acid (HNO3) had the role of pH regulator.

### 4.2. Sol–Gel Method

Basically, the necessary amounts of raw materials were determined starting from the composition of three calcium magnesium silicates, as follows: diopside (CaO·MgO·2SiO_2_, CMS_2_, Dy), akermanite (2CaO·MgO·2SiO_2_, C_2_MS_2_, Ak) and merwinite (3CaO·MgO·2SiO_2_, C_3_MS_2_, Mw). As a result, three oxide systems having variable content of calcium oxide (CaO) were established as targeted materials. Going to the experimental part, the measured volume of TEOS was solubilized in ethanol, the pH stabilized around 2 with HNO_3_, and the solution homogenized on a magnetic stirrer for 1 h. The nitrates were dissolved in distilled water by ultrasonication for 30 min. The two solutions were mixed and homogenized on a magnetic stirrer for another 1 h. Afterward, the final solution was kept at 60 °C, for 12 h, so as to allow the gelation and aging processes to take place, followed by drying at 80 °C for another 48 h. The dry gel was mortared and calcined at 600 °C so as to ensure the removal of the unwanted components: solvent molecules, organic fraction and nitrate groups; the calcination temperature was chosen based on the complex thermal analysis of all prepared gels (Figure 1). Furthermore, each composition was subjected to secondary thermal treatments: 800 and 1000 °C for Dy, 1000 and 1300 °C for Ak and Mw, for 2 h, with 10 °C/min heating rate and equilibrium cooling, resulting in the powders of interest. It was not possible to apply the same temperature values for all three calcined powders since Dy has a melting point of about 1390 °C, while Ak and Mw melt around 1450 °C; this means that heating up to 1300 °C makes Dy prone to melting, being well-known that the sol–gel method triggers a significant reduction of threshold temperatures (up to 200 °C). As a consequence, 1000 °C was maintained as maximum treatment temperature for Dy.

### 4.3. Materials Characterization

The dry gels were investigated from thermal and morphological points of view. The complex thermal analysis was recorded from room temperature to 1000 °C, with a rate of 5 °C/min, in air, on Shimadzu DTG-60 equipment (Shimadzu Corporation, Kyoto, Japan). The morphology was visualized by scanning electron microscopy (SEM), with a Quanta Inspect F microscope (FEI Company, Hillsboro, OR, USA) equipped with an energy-dispersive X-ray spectroscopy (EDX) probe; the operating parameters were: 30 kV accelerating voltage, 10 mm working distance and gold coating by DC magnetron sputtering for 40 s.

The thermally treated ceramics were assessed in terms of composition, structure and morphology. The elemental composition was determined with the help of the EDX probe, while the chemical bonds and groups were studied by Fourier transform infrared (FTIR) spectroscopy, with a Thermo Scientific Nicolet iS50 spectrophotometer (Thermo Fisher Scientific, Waltham, MA, USA), in the attenuated total reflection (ATR) mode; the working conditions were: 400–4000 cm^−1^ wavenumber range, 4 cm^−1^ resolution and 64 scans/sample. The phase composition and crystal structure were revealed by X-ray diffraction (XRD), with a Shimadzu XRD 6000 diffractometer (Shimadzu Corporation, Kyoto, Japan), using Ni-filtered Cu K*α* radiation (*λ* = 1.54 Å); the procedure involved: 10–60° 2*θ* range, 2°/min scan speed, 0.02° step size and 0.6 s preset time. To have a deeper view, a Rietveld refinement in HighScore Plus v3.0e software (Malvern Panalytical, Royston, UK) was applied for the powders thermally treated at the highest temperatures (1000 °C for Dy, 1300 °C for Ak and Mw); a polynomial function was employed for the background fit, with flat background coefficient, coefficient 1, coefficient 2, coefficient 3 and 1/x, a pseudo-Voigt profile function and Caglioti function for FWHM approximation. The samples crystallinity was evaluated through the intensity ratio of the diffraction peaks and of the sum of all measured intensity, as it is presented in Equation (3), where I_net_ is the net intensity (intensity of the crystalline peaks), I_tot_ is the total intensity and I_bgr_ is the background intensity (intensity which arises also for completely crystalline sample from imperfections of the sample).

Crystallinity = 100 × Σ I_net_/(Σ I_tot_ − Σ I_bgr_) (%) (3)

## Figures and Tables

**Table 1:** Composition of the ceramics corresponding to diopside (Dy); akermanite (Ak); and merwinite (Mw) compositions, thermally treated at the highest temperature (1000 or 1300 °C).

Phases/Sample	CMS_2_	C_2_MS_2_	C_3_MS_2_	C_2_S
	(wt%)			
**Dy-1000**	59.2	11.4	29.4	-
**Ak-1300**	25.4	26.5	**31.6**	16.5
**Mw-1300**	-	9.6	32.0	**58.4**

## References

[1] Ba Z., Chen Z., Huang Y., Feng D., Zhao Q., Zhu J., Wu D. (2018). Nanoporous diopside modulates biocompatibility, degradability and osteogenesis of bioactive scaffolds of gliadin-based composites for new bone formation. Int. J. Nanomed..

[2] Dasan A., Kraxner J., Grigolato L., Savio G., Elsayed H., Galusek D., Bernardo E. (2022). 3D printing of hierarchically porous lattice structures based on akermanite glass microspheres and reactive silicone binder. J. Funct. Biomater..

[3] Hafezi-Ardakani M., Moztarzadeh F., Rabiee M., Talebi A.R., Abasi-shahni M., Fesahat F., Sadeghian F. (2010). Sol-gel synthesis and apatite-formation ability of nanostructure merwinite (Ca_3_MgSi_2_O_8_) as a novel bioceramic. J. Ceram. Proc. Res..

[4] Han Z., Gao C., Feng P., Shen Y., Shuai C., Peng S. (2014). Silicon carbide whiskers reinforced akermanite scaffolds for tissue engineering. RSC Adv..

[5] Liu T., Deng Y., Gao C., Feng P., Shuai C., Peng S. (2015). Analysis of 3D printed diopside scaffolds properties for tissue engineering. Mater. Sci..

[6] Dasan A., Elsayed H., Kraxner J., Galusek D., Bernardo E. (2019). Hierarchically porous 3D-printed akermanite scaffolds from silicones and engineered fillers. J. Eur. Ceram. Soc..

[7] Han Z., Feng P., Gao C., Shen Y., Shuai C., Peng S. (2014). Microstructure, mechanical properties and in vitro bioactivity of akermanite scaffolds fabricated by laser sintering. Biomed. Mater. Eng..

[8] Shahrouzifar M.R., Salahinejad E. (2019). Strontium doping into diopside tissue engineering scaffolds. Ceram. Int..

[9] Pang S., Wu D., Kamutzki F., Kurreck J., Gurlo A., Hanaor D.A.H. (2022). High performing additively manufactured bone scaffolds based on copper substituted diopside. Mater. Des..

[10] Collin M.S., Venkatraman S.K., Mohana S., Sumathi S., Drweesh E.A., Elnagar M.M., Mosa E.S., Sasikumar S. (2021). Solution combustion synthesis of functional diopside, akermanite, and merwinite bioceramics: Excellent biomineralization, mechanical strength, and antibacterial ability. Mater. Today Commun..

[11] Ansari M., Malmir F., Salati A. (2020). Preparation and characterization of akermanite/merwinite scaffolds for bone tissue repair. J. Biomim. Biomater. Biomed. Eng..

[12] Razavi M., Fathi M., Savabi O., Vashaee D., Tayebi L. (2015). In vivo biocompatibility of Mg implants surface modified by nanostructured merwinite/PEO. J. Mater. Sci. Mater. Med..

[13] Hosseini Y., Emadi R., Kharaziha M. (2017). Surface modification of PCL-diopside fibrous membrane via gelatin immobilization for bone tissue engineering. Mater. Chem. Phys..

[14] Dong X., Li H., Lingling E., Cao J., Guo B. (2019). Bioceramic akermanite enhanced vascularization and osteogenic differentiation of human induced pluripotent stem cells in 3D scaffolds: In vitro and vivo. RSC Adv..

[15] Arastouei M., Khodaei M., Atyabi S.M., Nodoushan M.J. (2020). Poly lactic acid-akermanite composite scaffolds prepared by fused filament fabrication for bone tissue engineering. J. Mater. Res. Technol..

[16] Birhanu G., Doosti-Telgerd M., Zandi-Karimi A., Karimi Z., Daryasari M.P., Javar H.A., Seyedjafari E. (2022). Enhanced proliferation and osteogenic differentiation of mesenchymal stem cells by diopside coated Poly-L-lactic Acid-Based nanofibrous scaffolds. Int. J. Polym. Mater. Polym. Biomater..

[17] Nadernezhad A., Torabinejad B., Hafezi M., Baghaban-Eslaminejad M., Bagheri F., Najafi F. (2014). Poly (lactic-co-glycolic)/nanostructured merwinite porous composites for bone tissue engineering: Structural and in vitro characterization. J. Adv. Mater. Proc..

[18] Bafandeh M.R., Mojarrabian H.M., Doostmohammadi A. (2019). Poly (vinyl alcohol)/chitosan/akermanite nanofibrous scaffolds prepared by electrospinning. J. Macromol. Sci. B Phys..

[19] Ou J., Yin G.F., Zhou D.L., Chen X.C., Yao Y.D., Yang W.Z., Wu B.L., Xue M., Cui J., Zhu W.F. (2007). Preparation of merwinite with apatite-forming ability by sol-gel process. Key Eng. Mater..

[20] Shuai C., Han Z., Feng P., Gao C., Xiao T., Peng S. (2015). Akermanite scaffolds reinforced with boron nitride nanosheets in bone tissue engineering. J. Mater. Sci. Mater. Med..

[21] Nezafati N., Hafezi M., Zamanian A., Yasaei M., Mohammadi M.B. Preparation and characterization of a novel nano-structured merwinite scaffold prepared by freeze casting method. Proceedings of the 5th International Conference on Nanostructures.

[22] Venkatraman S.K., Choudhary R., Krishnamurithy G., Raghavendran H.R.B., Murali M.R., Kamarul T., Suresh A., Abraham J., Swamiappan S. (2021). Biomineralization, mechanical, antibacterial and biological investigation of larnite and rankinite bioceramics. Mater. Sci. Eng. C.

[23] Venkatraman S.K., Choudhary R., Krishnamurithy G., Raghavendran H.R.B., Murali M.R., Kamarul T., Suresh A., Abraham J., Praharaj S., Swamiappan S. (2022). Comparative investigation on antibacterial, biological and mechanical behaviour of monticellite and diopside derived from biowaste for bone regeneration. Mater. Chem. Phys..

[24] Reddy P.M., Lakshmi R., Dass F.P., Swamiappan S. (2014). Synthesis, characterization and formulation of sodium calcium silicate bioceramic for drug delivery applications. Sci. Eng. Compos. Mater..

[25] Yamamoto S., Kawamura N., Nonami T. (2019). Diopside synthesized by sol-gel method as phosphorus adsorption material: Evaluation of apatite deposition in pseudo body solution. Trans. Mater. Res. Soc. Jpn..

[26] Choudhary R., Koppala S., Swamiappan S. (2015). Bioactivity studies of calcium magnesium silicate prepared from eggshell waste by sol-gel combustion synthesis. J. Asian Ceram. Soc..

[27] Schumacher T.C., Volkmann E., Yilmaz R., Wolf A., Treccani L., Rezwan K. (2014). Mechanical evaluation of calcium-zirconium-silicate (baghdadite) obtained by a direct solid-state synthesis route. J. Mech. Behav. Biomed. Mater..

[28] Mohammadi H., Ismail Y.M.B., Shariff K.A., Noor A.F.M. (2019). Effect of substitutional strontium on mechanical properties of akermanite ceramic prepared by solid-state sintering. Mater. Today Proc..

[29] Sharafabadi A.K., Abdellahi M., Kazemi A., Khandan A., Ozada N. (2017). A novel and economical route for synthesizing akermanite (Ca_2_MgSi_2_O_7_) nano-bioceramic. Mater. Sci. Eng. C.

[30] Iwata N.Y., Tsunakawa S., Tanaka M., Utsu T., Matsumoto K. (1999). Improvements of apatite-forming abilities on pure and sodium-containing diopside substrates using porous diopside thin films as nucleating agent. MRS Online Proc. Libr..

[31] Gheisari Dehsheikh H., Karamian E. (2016). Characterization and synthesis of hardystonite (HT) as a novel nanobioceramic powder. Nanomed. J..

[32] Gheisari H., Karamian E., Soheily A. (2020). Survey and evaluation of merwinite (MW) as a new nanobioceramic powder. J. Nanoanal..

[33] Negrea R., Busuioc C., Constantinoiu I., Miu D., Enache C., Iordache F., Jinga S.I. (2019). Akermanite-based coatings grown by pulsed laser deposition for metallic implants employed in orthopaedics. Surf. Coat. Technol..

[34] Hafezi-Ardakani M., Moztarzadeh F., Rabiee M., Talebi A.R. (2011). Synthesis and characterization of nanocrystalline merwinite (Ca_3_Mg(SiO_4_)_2_) via sol-gel method. Ceram. Int..

[35] Iwata N.Y., Lee G.H., Tsunakawa S., Tokuoka Y., Kawashima N. (2004). Preparation of diopside with apatite-forming ability by sol-gel process using metal alkoxide and metal salts. Colloids Surf. B Biointerfaces.

[36] Lombardi M., Cacciotti I., Bianco A., Montanaro L. (2015). RKKP bioactive glass-ceramic material through an aqueous sol-gel process. Ceram. Int..

[37] Voicu G., Ene V.L., Sava D.F., Surdu V.A., Busuioc C. (2016). Sol-gel derived vitroceramic materials for biomedical applications. J. Non-Cryst. Solids.

[38] Duman S., Bulut B. (2021). Effect of akermanite powders on mechanical properties and bioactivity of chitosan-based scaffolds produced by 3D-bioprinting. Ceram. Int..

[39] Naga S.M., El-Maghraby H.F., Mahmoud E.M., Killinger A., Gadow R. (2019). Hydroxyapatite/diopside porous scaffolds: Preparation and in vitro study. Interceram.

[40] Bigham A., Hassanzadeh-Tabrizi S.A., Khamsehashari A., Chami A. (2018). Surfactant-assisted sol–gel synthesis and characterization of hierarchical nanoporous merwinite with controllable drug release. J. Sol-Gel Sci. Technol..

[41] Wu C., Chang J. (2004). Synthesis and apatite-formation ability of akermanite. Mater. Lett..

[42] No Y., Li J., Zreiqat H. (2017). Doped calcium silicate ceramics: A new class of candidates for synthetic bone substitutes. Materials.

[43] Nezafati N., Hafezi M., Zamanian A., Naserirad M. (2015). Effect of adding nano-titanium dioxide on the microstructure, mechanical properties and in vitro bioactivity of a freeze cast merwinite scaffold. Biotechnol. Prog..

[44] Hosseini Y., Emadi R., Kharaziha M., Doostmohammadi A. (2017). Reinforcement of electrospun poly(e-caprolactone) scaffold using diopside nanopowder to promote biological and physical properties. J. Appl. Polym. Sci..

[45] Shuai C., Liu T., Gao C., Feng P., Xiao T., Yu K., Peng S. (2016). Mechanical and structural characterization of diopside scaffolds reinforced with graphene. J. Alloys Compd..

[46] Teimouri A., Ghorbanian L., Dabirian I. (2016). Preparation and characterization of silk/diopside composite nanofibers via electrospinning for tissue engineering application. Int. J. Chem. Mol. Eng..

[47] Arastouei M., Khodaei M., Atyabi S.M., Nodoushan M.J. (2020). Improving the properties of the porous polylactic acid scaffold by akermanite nanoparticles for bone tissue engineering. J. Adv. Mater. Proc..

[48] Chen C., Watkins-Curry P., Smoak M., Hogan K., Deese S., McCandless G.T., Chan J.Y., Hayes D.J. (2015). Targeting calcium magnesium silicates for polycaprolactone/ceramic composite scaffolds. ACS Biomater. Sci. Eng..

[49] Wu C., Chang J., Zhai W., Ni S., Wang J. (2006). Porous akermanite scaffolds for bone tissue engineering: Preparation, characterization, and in vitro studies. J. Biomed. Mater. Res. B Appl. Biomater..

[50] Abdollahi M., Ghomi H. (2020). Fabrication of highly porous merwinite scaffold using the space holder method. Int. J. Mater. Res..

[51] Koppala S., John S.P., Balan R., Lokesh B., Munusamy S., Karthikeyan P., Godiya C.B., Chandragiri S.Y., Aminabhavi T.M., Duan K. (2022). Glowing combustion synthesis, characterization and biomedical properties of Sr-hardystonite (Sr_2_ZnSi_2_O_7_) powders. Ceram. Int..

[52] Jinga S.I., Constantinoiu I., Surdu V.A., Iordache F., Busuioc C. (2019). Sol-gel-derived mineral scaffolds within SiO_2_–P_2_O_5_–CaO–MgO–ZnO–CaF_2_ system. J. Sol.-Gel. Sci. Technol..

[53] Jinga S.I., Anghel A.M., Brincoveanu S.F., Bucur R.M., Florea A.D., Saftau B.I., Stroe S.C., Zamfirescu A.I., Busuioc C. (2021). Ce/Sm/Sr-incorporating ceramic scaffolds obtained via sol-gel route. Materials.

[54] Prefac G.A., Milea M.L., Vadureanu A.M., Muraru S., Dobrin D.I., Isopencu G.O., Jinga S.I., Raileanu M., Bacalum M., Busuioc C. (2020). CeO_2_ containing thin films as bioactive coatings for orthopaedic implants. Coatings.

[55] Draghici D.A., Mihai A.A., Aioanei M.O., Negru N.E., Nicoara A.I., Jinga S.I., Miu D., Bacalum M., Busuioc C. (2022). Strontium-substituted bioactive glass-ceramic films for tissue engineering. Bol. Soc. Esp. Ceram. Vidr..

[56] Schitea R.I., Nitu A., Ciobota A.A., Munteanu A.L., David I.M., Miu D., Raileanu M., Bacalum M., Busuioc C. (2020). Pulsed laser deposition derived bioactive glass-ceramic coatings for enhancing the biocompatibility of scaffolding materials. Materials.

